# Patient safety in out-of-hours primary care: a review of patient records

**DOI:** 10.1186/1472-6963-10-335

**Published:** 2010-12-10

**Authors:** Marleen Smits, Linda Huibers, Brian Kerssemeijer, Eimert de Feijter, Michel Wensing, Paul Giesen

**Affiliations:** 1 Scientific Institute for Quality of Healthcare, Radboud University Nijmegen Medical Centre, Nijmegen, the Netherlands

## Abstract

**Background:**

Most patients receive healthcare in primary care settings, but relatively little is known about patient safety. Out-of-hours contacts are of particular importance to patient safety. Our aim was to examine the incidence, types, causes, and consequences of patient safety incidents at general practice cooperatives for out-of-hours primary care and to examine which factors were associated with the occurrence of patient safety incidents.

**Methods:**

A retrospective study of 1,145 medical records concerning patient contacts with four general practice cooperatives. Reviewers identified records with evidence of a potential patient safety incident; a physician panel determined whether a patient safety incident had indeed occurred. In addition, the panel determined the type, causes, and consequences of the incidents. Factors associated with incidents were examined in a random coefficient logistic regression analysis.

**Results:**

In 1,145 patient records, 27 patient safety incidents were identified, an incident rate of 2.4% (95% CI: 1.5% to 3.2%). The most frequent incident type was treatment (56%). All incidents had at least partly been caused by failures in clinical reasoning. The majority of incidents did not result in patient harm (70%). Eight incidents had consequences for the patient, such as additional interventions or hospitalisation. The panel assessed that most incidents were unlikely to result in patient harm in the long term (89%). Logistic regression analysis showed that age was significantly related to incident occurrence: the likelihood of an incident increased with 1.03 for each year increase in age (95% CI: 1.01 to 1.04).

**Conclusion:**

Patient safety incidents occur in out-of-hours primary care, but most do not result in harm to patients. As clinical reasoning played an important part in these incidents, a better understanding of clinical reasoning and guideline adherence at GP cooperatives could contribute to patient safety.

## Background

Patient safety has become a priority in the past decade, and many Western countries have examined the rate of adverse events in hospital care [1-8]. A systematic review reported that in 9.2% of all hospital admissions one or more adverse events (incidents with patient harm) occurred, while nearly half (43.5%) of these could have been prevented and 7.4% contributed to death [9].

Up to now, the focus in patient safety has mainly been on hospital care. Most patients, however, receive their healthcare in primary care settings, particularly in countries with a strong primary care system [10,11]. Relatively little is known about patient safety in these settings. In a previous review, the rate of incidents in primary care has been estimated as ranging between 5 and 80 in 100,000 consultations [12]. However, the majority of the included studies used event reporting by professionals as their research method. Reporting systems considerably under-report patient safety events and are not appropriate for estimating incidence rates [13,14]. A recent study found evidence to suggest that the rate is much higher. This record review study reported that an incident with harm occurred every 48 consultations [15].

The literature on incidents in primary care so far has not included out-of-hours primary care. Many patient contacts with primary care occur out-of-hours. In the Netherlands, out-of-hours primary care is organised by general practitioner (GP) cooperatives involving 40 to 250 GPs. GPs working in such cooperatives are registered GPs who work in primary care practices in daytime. The GP cooperative is intended to deal with urgent requests for medical attendance that cannot wait until the next day; it is available daily from 5 pm to 8 am on weekdays and the entire weekend (see Table 1 for more characteristics). Patient safety is of particular importance in GP cooperatives because of the high patient throughput and the diversity of clinical conditions presented, which are more likely to be urgent than in daytime primary care. Identification of medical urgency during telephone contacts with GP cooperatives proved to be suboptimal [16-18]. GPs and nurses working in cooperatives treat patients they do not know and have to decide on advice and medical treatment with limited knowledge of the patients' medical history. In addition, GPs work in shifts and have to collaborate with other healthcare providers, which increases the risk of errors caused by discontinuity in information transfer [16,17].

**Table 1:** Features of GP cooperatives in the Netherlands 161718.

GP cooperative
Out-of-hours is defined as daily from 5 pm to 8 am and the entire weekend
Population of 100,000 to 500,000 patients
Distances up to 30 km
GP cooperative usually situated near a hospital
Access through a single regional telephone number
Telephone triage by nurses
50-250 GPs, on call 4 hours a week on average
GP shift of 6 to 8 hours
Per shift, GPs have different roles: home visits, centre consultations, and supervising telephone triage
Drivers in identifiable GP cars that are fully equipped (e.g., O_2_, i.v. drip equipment, automated external defibrillator, medication)
Information and communication technology (ICT) support, including electronic patient files and online connection to the GP car

The aim of our study was to gain an understanding of the incidence, types, causes, and consequences of patient safety incidents in out-of-hours GP cooperatives. A secondary aim was to examine factors that are associated with the occurrence of patient safety incidents.

## Methods

### Study design and setting

In 2009, a retrospective patient record review study was performed to examine patient safety incidents in GP cooperatives providing out-of-hours primary care in the Netherlands. Data were collected in a sample of general practices associated with selected GP cooperatives, as general practices keep complete documentation of the healthcare their patients received, including their contacts with GP cooperatives and subsequent healthcare use.

Four GP cooperatives were selected. In this selection, we aimed to provide a good reflection of the national situation by taking into account location in the country, degree of urbanisation, and size of the cooperatives. A convenience sample of seventeen general practices linked to these GP cooperatives were invited to participate in the study, sixteen of which agreed to participate.

For each of the four GP cooperatives, we selected a sample of at least 250 patients who had contacted the GP cooperative in April or May 2009. For each GP cooperative, the first 250 contacts that were eligible for review were included in the study (consecutive sampling). A 'contact' was a patient who visited the GP cooperative, received telephone advice from the cooperative, or received a home visit from a GP working for the cooperative. For patients with multiple contacts with a GP cooperative within the study period, only the first contact was included in the sample as the index contact. We excluded administrative reports without a patient contact with the GP cooperative (for example, a note from a hospital reporting a patient's demise).

Several measures were taken to ensure the confidentiality of the information we collected. No patient or physician names were included in the database, and reviewers and researchers (study staff) signed a confidentiality agreement to maintain the confidentiality of the information. The Arnhem-Nijmegen ethical committee waived approval for this study.

### Patient record review procedure

**Figure 1:**
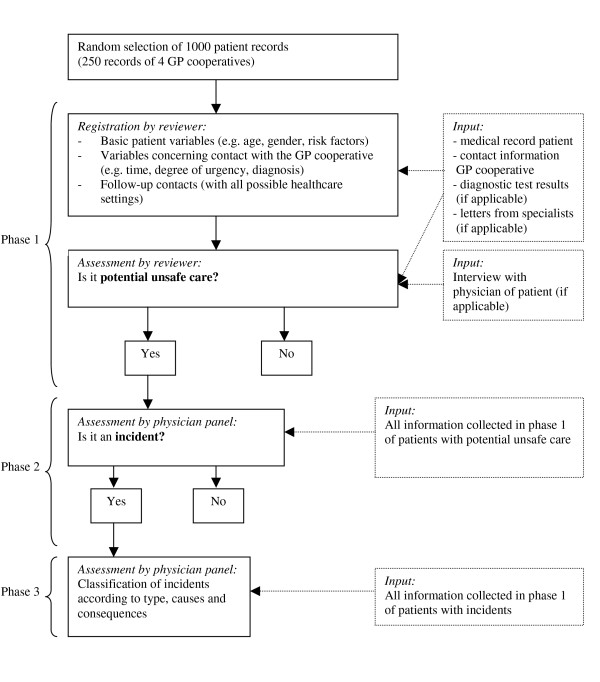
**Record review procedure**.

The record review procedure consisted of three phases (Figure 1). In the first phase, reviewers assessed the medical records of all sampled patients. The reviewers were an experienced GP and a medical student in his final year. They used a review form that had been developed for the study and was based on a form used for incident reporting in general practice and checked for face validity by an expert panel. The review procedure was pre-tested on 100 patient records in one general practice, resulting in a few small alterations to the review form. The pre-tested records were reviewed once more, using the adapted review form, and were included in the study.

The reviewers assessed the patient contacts in the general practices. The general practices supplied the reviewers with the electronic medical records of sampled patients, including information about the contact with the GP cooperative, test results, and specialists' letters. Reviewers assessed whether one or more potential patient safety incidents had occurred during the patient's contact with the GP cooperative. A patient safety incident was defined as an unintended event during the care process that resulted, could have resulted or still might result in harm to the patient [19].

Firstly, the reviewers registered some basic patient characteristics: age, gender, whether the patient had contacted his GP about the same health problem within one week before contacting the GP cooperative, and whether the patient was a high-risk patient. Patients with cardiac and vascular disease, diabetes mellitus, asthma/COPD, polypharmacy (> 5 medications), immune system disease, malignancy (active), pregnancy, or a combination of these conditions, were defined as high-risk patients.

Secondly, the reviewers registered measures concerning the patients' contact with the GP cooperative: contact type, contact time, documented degree of urgency, reason for contact, diagnosis, and medical treatment. For a period of at least four months after the contact, moreover, reviewers registered whether patients had follow-up contacts (with a GP, GP cooperative, or hospital casualty department) relating to the index health problem, whether patients were admitted to a hospital, or whether patients died. Finally, the reviewers assessed whether the healthcare provided at the GP cooperative was potentially unsafe [20]. If the reviewers had doubts about a particular case, the patient's GP was interviewed to clarify what had happened. If the reviewers signalled potentially unsafe healthcare, the patient's medical record proceeded to phase two.

In phase two, the medical records of patients who had received potentially unsafe healthcare were discussed by a panel of physicians to determine if a patient safety incident had indeed occurred. This panel discussed the potential incidents until consensus was achieved. Besides the two reviewers, the panel consisted of two experienced physicians.

In the third and final phase, the physician panel tentatively classified the incidents according to type, causes, and consequences (Table 2). Six types of incidents were distinguished: 'organisation', 'communication', 'diagnosis', 'treatment', 'prevention', and 'triage'. The first four types were derived from a classification model that had been developed for and is commonly used in primary care [21]. Causes of the incidents were analysed using the Eindhoven Classification Model (ECM) of the PRISMA-method,[22,23] which has proved to be a reliable tool [24] and has been used as a foundational component in the conceptual framework for the International Classification for Patient Safety (ICPS) of the World Health Organization (WHO) and its World Alliance for Patient Safety programme [25,26].

**Table 2:** Overview of classifications

Subject	Categories
**Type of patient safety incidents **[21]	Organisation
	Communication
	Prevention
	Triage
	Diagnostics
	Treatment
**Cause(s) of patient safety incident **[22,23]	Technical (Design; Construction; Materials; External)
	Organisational (Transfer of knowledge; Protocols; Management priorities; Culture; External)
	Human (Clinical reasoning/Knowledge-based behaviour; Qualifications; Coordination; Verification; Intervention; Monitoring; Slips; Tripping; External)
	Patient-related
	Other
**Harm to the patient **[27]	Error, but no harm
	Error resulting in harm to the patient (Emotional harm; Temporary harm; Initial/prolonged hospitalisation; Permanent harm; Intervention to sustain a patient's life)
	Error resulting in death
	Error, but harm indeterminate
**Probability of severe harm or death**	Very likely
	Likely
	Not likely

The consequences of the incidents were classified, using the 'severity of outcome' dimension of the International Taxonomy of Medical Errors in Primary Care [27]. In addition, the probability of (severe) harm in the future was assessed (very likely, likely, not likely).

### Inter-rater reliability

The first ten patient records from five general practices (N = 50) were independently assessed by the two reviewers to determine their agreement on the presence or absence of potentially unsafe healthcare.

### Statistical analysis

Study results were first described using descriptive statistics and frequency tables. To test which patient and contact factors predicted the occurrence of patient safety incidents, univariate multilevel logistic regression analyses and a forward stepwise multilevel logistic regression analysis were performed, using GP cooperative as random factor in the model (PROC GLIMMIX). Results were considered statistically significant at p < 0.05. Data were analysed using SPSS 15.0 and SAS 9.2.

## Results

### Description of patient safety incidents

#### Incidence and types

A total of 1,145 patient records were reviewed (for practical reasons, 248, 328, and 319 records were reviewed in three GP cooperatives and 250 records in the fourth cooperative). Agreement between the two reviewers was 98.0% and Cohen's kappa was 0.79 (95% CI: 0.39 to 1.00). The quality of the patient records was predominantly judged as good (94%). In 1,145 patient records, reviewers identified 56 potential patient safety incidents. The physician panel judged 27 of these to be patient safety incidents, which is an incident rate of 2.4% (95% CI: 1.5% to 3.2%).

Three incidents were related to more than one incident type. The most frequent incident type was treatment: patients receiving inadequate or suboptimal treatment (N = 15; 56%). Nine incidents (33%) were related to triage, meaning the urgency or care of patients' complaints had not been correctly assessed either by the triage nurse or by the supervising physician. Six incidents (22%) were related to diagnosis: misguided diagnostic reasoning or wrong diagnoses (see Table 3 for examples).

**Table 3:** Examples of patient safety incidents

Incident type	Study example
Treatment	A patient visited the GP cooperative with a metal foreign body in his eye. GP removed splinter and sent patient home. After four hours, the patient called the GP cooperative because of increasing pain in the eye. At patient's initial visit, no eye antibiotic had been prescribed nor had a bandage been applied.
	
	Patient visited GP cooperative with dog bite in hand. No antibiotics were administered.
	
Triage	Patient called GP cooperative with pain in left side of abdomen. Patient had had diverticulitis before and asked for antibiotics. Patient received this medication prescription by telephone.
	
	Patient called GP cooperative with acute loss of hearing in one ear. Assistant told patient over the telephone that it was a cerumen impaction. Later, the patient got an urgent referral to an ear, nose, and throat specialist, and it turned out to be sudden deafness.
	
Diagnosis	A 22-year-old patient presented herself at the GP cooperative with acute pain in the left thorax without fever. The GP heard chest crepitations and diagnosed the patient with pneumonia. The patient was treated with antibiotics. After six days, the patient visited her own GP because of persisting pain. The GP sent the patient out for an X-ray. The chest X-ray showed a pneumothorax, which could have been treated conservatively.
	
	A four-year-old patient visited the GP cooperative with a foot injury after being jammed between the spokes of her mother's bicycle. Her foot was blue, and she could not walk or stand on it. The GP did not perform a physical examination. Diagnosis: no fracture. Wait-and-see policy. Later the patient turned out to have epiphysiolysis.

#### Causes

For 27 patient safety incidents, a total of 30 causes could be identified. The causal factors fell into three different categories: clinical reasoning, protocols, and patient-related factors. All incidents had at least partly been caused by failures in clinical reasoning: the inability of individuals to apply their existing knowledge to a novel situation. In two cases, a patient-related factor was relevant, that is, a failure related to patient characteristics or conditions that are beyond the control of staff. In one case, the absence of an adequate protocol contributed to the incident. No technical causal factors were identified.

#### Actual and potential consequences

The majority of patient safety incidents did not result in actual patient harm (N = 19; 70%). Eight incidents had consequences for patients: an extra intervention was needed in six cases, and two patients had to be admitted to a hospital. No incidents resulted in permanent harm or death. Most incidents were not likely to result in patient harm in the long term (N = 24; 89%). In three cases (11%), future consequences were possible but not likely.

### Factors associated with the occurrence of patient safety incidents

Table 4 presents an overview of contact and patient characteristics for the total sample of contacts and the subsample of contacts with patient safety incidents. Univariate analyses showed that age and being a high-risk patient were positively related to incident rate. The patients' gender, preceding contacts with patients' own GP, type and time of contact, and the urgency of the contact were not related to the incident rate.

**Table 4:** Overview of patient and contact characteristics and incident rate in each category

Variable		Number of contacts (%) N = 1145	Number of incidents (%) N = 27	Incident rate (per 100 contacts)^3^
**Gender**	Male	520 (45.4)	13 (48.1)	2.5
	Female	625 (54.6)	14 (51.9)	2.2
				
**High-risk patient^1^**	Yes	307 (26.8)	14 (51.9)	4.6
	No	838 (73.2)	13 (48.1)	1.6
				
**Earlier contact with their own GP^2^**	Yes	139 (12.1)	5 (18.5)	3.6
	No	1006 (87.9)	22 (81.5)	2.2
				
**Type of contact**	Telephone advice	412 (36)	10 (37.0)	2.4
	Consultation at GP cooperative	629 (54.9)	13 (48.1)	2.1
	Home visit	104 (9.1)	4 (14.8)	3.8
				
**Time of contact**	Day (8 am - 5 pm)	495 (43.2)	11 (40.7)	2.2
	Evening (5 - 11 pm)	519 (45.3)	10 (37.0)	1.9
	Night (11 pm - 8 am)	129 (11.3)	6 (22.2)	4.7
	Missing	2 (0.2)	0 (0)	0
				
**Degree of urgency**	U1 (life-threatening, immediate care)	14 (1.2)	0 (0)	0
	U2 (acute, evaluation within one hour)	57 (5.0)	1 (3.7)	1.8
	U3 (urgent, evaluation within a few hours)	393 (34.3)	13 (48.1)	3.3
	U4 (routine, no time pressure)	681 (59.9)	13 (48.1)	1.9
				
***Total***		*1145*	*27*	*2.4*

^1^High-risk patients were defined as patients with one or more of the following conditions: cardiac and vascular disease, diabetes mellitus, asthma/COPD, polypharmacy (> 5 medications), immune system disease, malignity (active), pregnancy.

^2^Earlier was defined as within two weeks prior to the index contact.

**^3^**The incident rates did not differ significantly in the multivariate regression analysis.

The mean age in the total sample was 36.6 years (SD = 24.8). The mean age was 52.2 years (SD = 23.5) for patients with incidents, compared to 36.2 years (SD = 24.8) for patients without incidents. Multilevel analyses showed that the likelihood of an incident increased with 1.03 for each year of advancing age (95% CI: 1.01 to 1.04). The likelihood of an incident was 3.05 times higher for high-risk patients than for normal-risk patients (95% CI: 1.42 to 6.58). In the stepwise multilevel logistic regression, only the effect of age was significant.

## Discussion

### Main findings

Patient safety incidents occurred in 2.4% of all patient contacts with GP cooperatives (95% CI: 1.5% to 3.2%). The incidents identified in our study were related to triage, diagnosis, or treatment. They were all (at least partly) caused by failures in clinical reasoning. The majority of the incidents did not result in (permanent) harm to patients, but a few incidents were associated with temporary harm. With increasing patient age, an incident's probability of occurrence increased significantly.

### Strengths and limitations

Our study was based on a large sample of patient contacts. We performed our study in four GP cooperatives that were spread across the Netherlands and varied in size and degree of urbanisation because we pursued a nationally representative study. GP practices were a convenience sample. Although our unit of analysis was patient contacts with multiple GPs taking shifts at four GP cooperatives, we cannot exclude a risk of selection bias.

Data were collected in general practices associated with the GP cooperatives. This enabled reviewers to study not only the contact information of the GP cooperative, but also the medical records of the patients' own GPs, which include information about patients' healthcare use after they contacted the GP cooperative.

Descriptive retrospective analyses depend on data quality. Some details of patient contacts are not written down, especially in case of telephone contacts [28,29]. The patient record reviewing method was carefully developed and tested, and proved to have high inter-rater reliability. However, no method for identifying incidents is perfect,[13,14] and a combination of methods has been recommended [30,31]. The study design we used will likely have enabled us to discover severe incidents but might have led us to underestimate the number of minor incidents without consequences and incidents in particular domains, such as accessibility issues.

The number of causes identified per incident was small. It was difficult to obtain information on all contributing factors just by reading patient records. Interviewing people who were involved in the incidents would have produced a more complete view of the causal factors involved. However, confidentiality clauses made it impossible for us to do so in our study.

Furthermore, the majority of causes were human. Healthcare providers may be less inclined to note down technical or organisational factors in the medical records of individual patients, which might explain the small numbers of technical and organisational factors identified.

Finally, the study period was two months (April and May 2009). As the quantity and variety of health problems presented by patients vary with time of year, a study covering an entire year might have provided a different perspective.

### Interpretation of the results

The results show that patient safety incidents do occur in out-of-hours primary care but that most do not result in harm to patients. However, as there are large numbers of patient contacts, the accumulated effect of the incidents could be significant.

No earlier studies have examined incidents in GP cooperatives or similar out-of-hours healthcare settings. In a review of patient safety in daytime primary care, the incidence rate was estimated between 0.005% and 0.8% [12], which is much lower than the rate found in our study (2.4%). The research methods used may have contributed to this discrepancy: most studies in the review used event reporting, whereas we used patient record review. Furthermore, our definition of patient safety incidents was broad and included more than just events that resulted in patient harm. Our findings are comparable to patient record review studies into incidents in daytime primary care reporting incidence rates of 2.1% [15] and 2.5% (Gaal, personal communication, 2010). These findings suggest that patient safety in out-of-hours primary care is comparable to that in daytime primary care, even though the symptoms presented and medical treatments provided differ substantially between those settings.

Failures in clinical reasoning played a part in all incidents in our study. These failures might be related to the fact that GPs in GP cooperatives do not know patients and must decide on medical treatment without having full knowledge of patients' medical history. In addition, they have virtually no possibilities for diagnostic examinations such as X-rays and laboratory tests without referral to a specialist.

We found that the probability of experiencing an incident was higher for older patients (OR = 1.03) but that this did not apply to high-risk patients. This result was unexpected because co-morbidity was believed to be the explanation for higher age-related probability. Deafness or cognitive disorders in older patients might impede communication, which makes clinical reasoning, diagnosis, and treatment more difficult for GPs. A study by Sari et al. (2008) in an NHS hospital also found that, with increasing age, there was a raised risk of experiencing an adverse event (OR = 1.03) [32].

Finally, nurse telephone triage has been adopted to reduce GP workload, but its safety is under debate [33-36]. Our study did not find an increased risk of incidents in patients that received telephone advice as compared to consultation at the clinic or home visits.

### Recommendations for practice

Information about incidents can be used to develop interventions to improve patient safety. It is recommended for out-of-hours healthcare services to examine incidents by way of periodic patient contact audits, including follow-up information from the patients' own GPs, and to combine these with incident reporting by professionals. Incidents should be discussed with professionals to improve clinical reasoning and to reduce the likelihood of reoccurrence. GPs should adhere to guidelines and document their reasons for deviating from these guidelines.

Moreover, system-wide interventions are suggested to enhance patient safety in primary care, such as developing leadership and culture supporting safety behaviour; constructing barriers in systems to prevent human error; and using sensible technology, such as meaningful warnings, communication, and knowledge coupling [37].

Finally, existing national clinical guidelines may be less applicable to out-of-hours primary care. Relevant topics related to acute medical problems need to be elaborated [38].

### Recommendations for future research

We need to improve our understanding of the reasons why GPs in out-of-hours healthcare make errors in clinical reasoning, as these are the most frequent causes of patient safety incidents. Lack of information on patients' medical history, insufficient medical knowledge, and high workload could all play a part. Further research into the underlying mechanisms of failures in clinical reasoning is recommended.

Because the number of patients actually suffering harm from incidents is small, actual harm is not a good outcome measure for evaluating the effectiveness of patient safety interventions, such as evaluating the implementation effectiveness of clinical guidelines. Outcome measures such as unnecessary prolongation or aggravation of symptoms will be more valuable for evaluating prevention strategies. We recommend, therefore, that future research should include this broader view of patient safety.

## Conclusions

In this study, we found that patient safety incidents occurred in 2.4% of all patient contacts with GP cooperatives. The incidents identified were related to triage, diagnosis, or treatment and were all (at least partly) caused by failures in clinical reasoning. Most incidents did not result in harm to patients. With increasing patient age, an incident's probability of occurrence increased significantly.

Our findings report a relatively low incident rate at GP cooperatives in the Netherlands. As there are large numbers of contacts and patients, however, the accumulated effect of the incidents on patient well-being and the healthcare delivery system could be significant. Moreover, as clinical reasoning played an important part in these incidents, further understanding of clinical reasoning and guideline adherence at GP cooperatives could contribute to healthcare safety.

## Competing interests

The authors declare that they have no competing interests.

## Authors' contributions

MS contributed to data analysis and wrote the manuscript. LH contributed to the data collection process and the writing of the manuscript. BK collected and analysed data and contributed to the writing of the manuscript. EdF collected data and critically read the manuscript. MW and PG designed the study, supervised data collection, contributed to data interpretation, and critically revised the manuscript for intellectual content. MW arranged funding. All authors approved the final version of the manuscript.

## Funding

The study presented in this paper was part of a large study on patient safety in primary care in the Netherlands. The Dutch Ministry of Health, Welfare and Sport (VWS) initiated and funded the project (without restrictions on the scientific work; grant number 313741).

## Pre-publication history

The pre-publication history for this paper can be accessed here:

http://www.biomedcentral.com/1472-6963/10/335/prepub
